# Application of kernel principal component analysis and computational machine learning to exploration of metabolites strongly associated with diet

**DOI:** 10.1038/s41598-018-20121-w

**Published:** 2018-02-21

**Authors:** Yuka Shiokawa, Yasuhiro Date, Jun Kikuchi

**Affiliations:** 1 RIKEN Center for Sustainable Resource Science, 1-7-22 Suehiro-cho, Tsurumi-ku, Yokohama 235-0045 Japan; 2 Graduate School of Medical Life Science, Yokohama City University, 1-7-29 Suehiro-cho, Tsurumi-ku, Yokohama 230-0045 Japan; 3 Graduate School of Bioagricultural Sciences and School of Agricultural Sciences, Nagoya University, 1 Furo-cho, Chikusa-ku, Nagoya 464-8601 Japan

## Abstract

Computer-based technological innovation provides advancements in sophisticated and diverse analytical instruments, enabling massive amounts of data collection with relative ease. This is accompanied by a fast-growing demand for technological progress in data mining methods for analysis of big data derived from chemical and biological systems. From this perspective, use of a general “linear” multivariate analysis alone limits interpretations due to “non-linear” variations in metabolic data from living organisms. Here we describe a kernel principal component analysis (KPCA)-incorporated analytical approach for extracting useful information from metabolic profiling data. To overcome the limitation of important variable (metabolite) determinations, we incorporated a random forest conditional variable importance measure into our KPCA-based analytical approach to demonstrate the relative importance of metabolites. Using a market basket analysis, hippurate, the most important variable detected in the importance measure, was associated with high levels of some vitamins and minerals present in foods eaten the previous day, suggesting a relationship between increased hippurate and intake of a wide variety of vegetables and fruits. Therefore, the KPCA-incorporated analytical approach described herein enabled us to capture input–output responses, and should be useful not only for metabolic profiling but also for profiling in other areas of biological and environmental systems.

## Introduction

Innovation in computer-based technology has caused not just advancements of computer-associated technology but also considerably contributions of their ripple effects to technological progress in research fields of chemistry and biology. This technological innovation facilitates advancements in sophisticated and diverse analytical instruments, enabling massive amounts of data collection with relative ease. The increasing opportunity of handling “big data” has accompanied with a fast-growing demand for technological progress in highly analytical methods for mining “big data.” From this viewpoint, machine learning approaches such as deep learning and data mining techniques are currently being developing at a fast clip.

One research field in chemistry and biology that acquires and handles “big data” is metabolomics or metabolic profiling. A massive amount of data in metabolomics studies is typically obtained by nuclear magnetic resonance (NMR) spectroscopy and mass spectrometry. Especially, NMR spectroscopy is a non-destructive method for measurements of complex metabolites derived from biological systems^1–3^. In addition, NMR has advantages for analytical reproducibility and inter-convertibility among different institutions^4,5^. Therefore, NMR-based metabolic profiling has been applied to various biological and environmental samples^6–13^. These types of research benefit from several useful and helpful databases and analytical support tools for preprocessing of spectral data and assignments of metabolites in complex chemical mixtures in NMR-based metabolic profiling. Such databases and tools, for example, include the human metabolome database^14^, biological magnetic resonance data bank^15^, ^1^H (^13^C) TOCCATA^16,17^, SpinAssign^18^, SpinCouple^19^, MetaboAnalyst^20^, NMRShiftDB^21^, MVAPACK^22^, rNMR^23^, BATMAN^24^, statistical total correlation spectroscopy^25^, fragment-assembly approach^26^, and signal enhancement by spectral integration (SENSI) method^27^.

In the fields of NMR-based metabolomics, one key multivariate analysis is principal component analysis (PCA). PCA is an unsupervised method and a kind of “linear” multivariate analyses. Although PCA is able to capture meaningful tendencies in some datasets, PCA is not a panacea for analyses in all instances. For example, some cases in metabolic variations have typically non-linear relationships. Therefore, general PCA is not able to capture “non-linear” metabolomic relationships from various metabolic reactions in living organisms. Therefore, advances in powerful data mining methods that would allow the discoveries of valuable information from massive datasets have been eagerly anticipated. To circumvent difficulties in obtaining valuable information that cannot be extracted by conventional linear PCA methods, we focused on “non-linear” kernel PCA (KPCA)^28^. KPCA is an enhanced PCA method that incorporates a kernel function, thereby facilitating solution of non-linear problems. KPCA was previously applied to analysis of NMR-based metabolic profiling^29^. However, KPCA is limited by an inability to determine importance of variables in contrast to linear PCA where it is possible to identify key variables that contribute to PCA score profiles. Thus, it was important to overcome this limitation for evaluation of key metabolites and for discovery of useful biomarkers in NMR-based metabolic profiling studies. To identify key variables for kernel-based methods, several variable selection approaches have been previously reported in supervised classifications and regressions^30,31^, however, determination of important variables for unsupervised data using kernel-based methods is still challenging.

**Figure 1:**
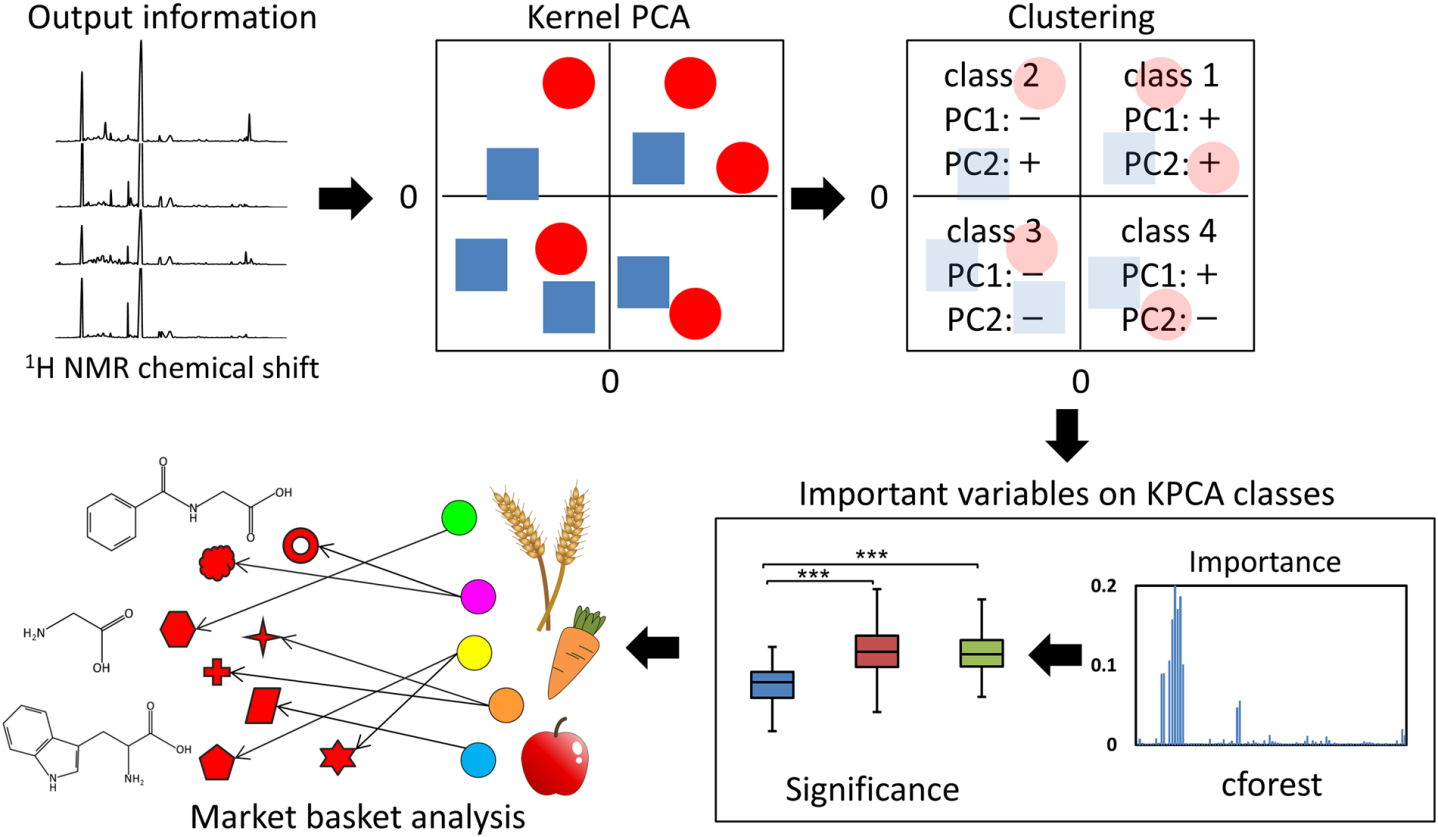
Analytical flow of the study. Kernel principal component analysis (KPCA) was calculated from urinary organic (nuclear magnetic resonance) and inorganic (inductively coupled plasma optical emission spectrometry) data. Subsequently, cforest was used to identify significant metabolites in four groups generated from the KPCA results. Finally, market basket analysis was used to obtain human lifestyle-associated information related to significant metabolites. The colored circles and red symbols in the market basket analysis indicate individual nutrients and metabolites (for the purposes of illustration), respectively. The image was drawn by Yuka Shiokawa.

Here, we describe a KPCA-incorporated analytical approach for the extraction of useful information from NMR-based metabolic profiling datasets. To overcome the limitation concerning important variable identifications in unsupervised KPCA, we incorporated a random forest conditional variable importance measure (cforest)^32^, a form of machine learning, into the KPCA-based analytical approach to determine the importance of variables. The obtained importance was validated using statistical tests and further analyzed using a market basket analysis (MBA)^33^ to evaluate input–output responses (urinary metabolites and minerals associated with dietary food and nutritional information) in humans (Fig. 1).

## Results and Discussion

### Non-linear KPCA

**Figure 2:**
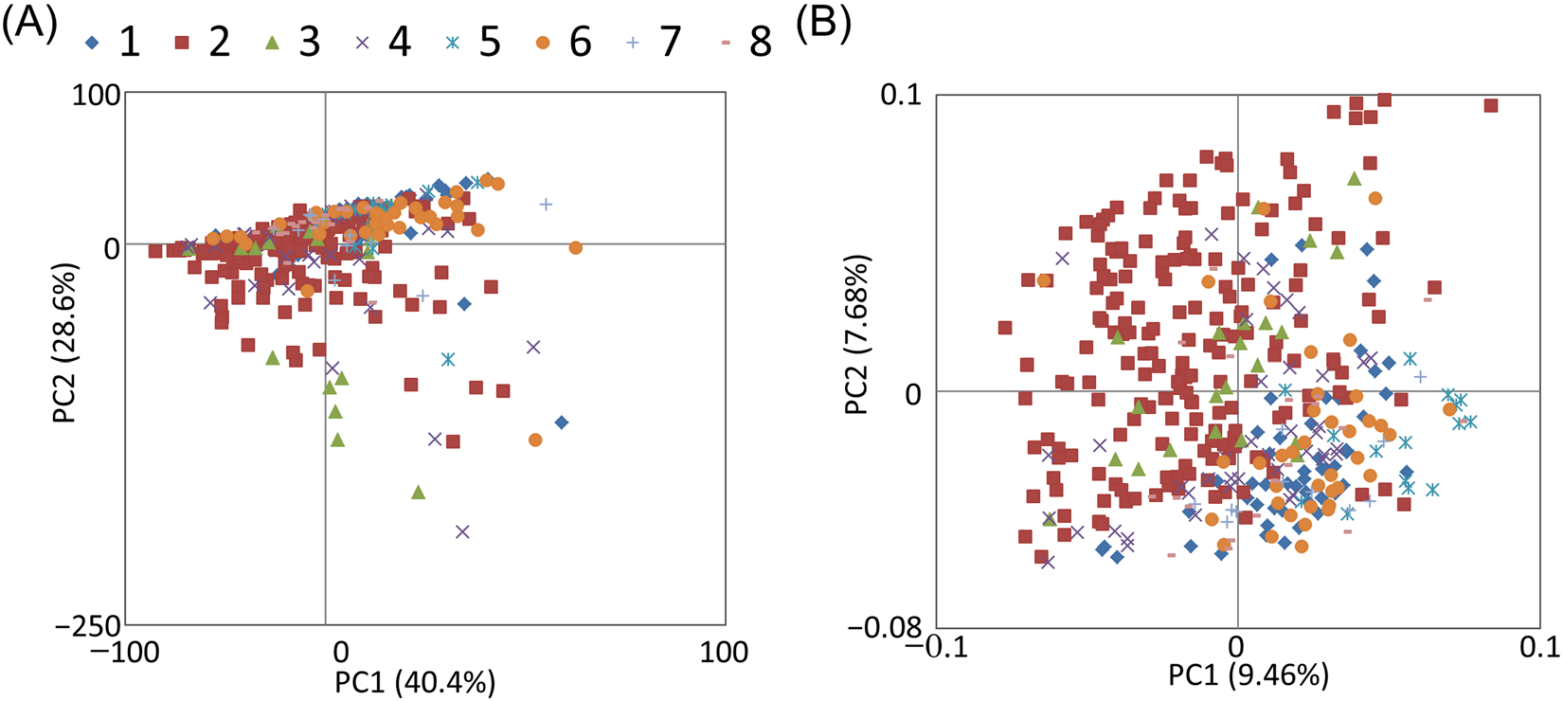
Comparison of profiles from conventional principal component analysis (PCA) (**A**) and kernel PCA (**B**). The symbols and numbers indicate individual subjects.

In this study, urinary metabolic and elemental data obtained from NMR and inductively coupled plasma optical emission spectrometry (ICP-OES), respectively (Figure S1), were integrated on a data matrix prior to KPCA. KPCA was performed using the analysis of variance (ANOVA) kernel function after changing the sigma parameter from 0.05 to 0.3 with the degree parameter d = 1 (Figure S2), 2, and 3 (data not shown). Using the PC1 contribution rate from KPCA, we determined the sigma value (0.135) with the parameter d = 1 as the KPCA parameters for further analyses. We used these determined parameters to demonstrate that KPCA and conventional PCA yielded different profiles (Fig. 2). With conventional PCA, many samples were concentrated at particular positions on the score plot; therefore, it was difficult to identify any characteristics among samples. In contrast, with KPCA, the samples were holistically dispersed over the score plot, and the profiles tended to cluster according to individual differences. In this study, dispersion on the scores plot is very important to avoid biased grouping and generation of an unbalanced dataset. Thus, KPCA compared to PCA was suitable to use in subsequent analyses.

### Important variable identifications of KPCA by incorporation of cforest

**Figure 3:**
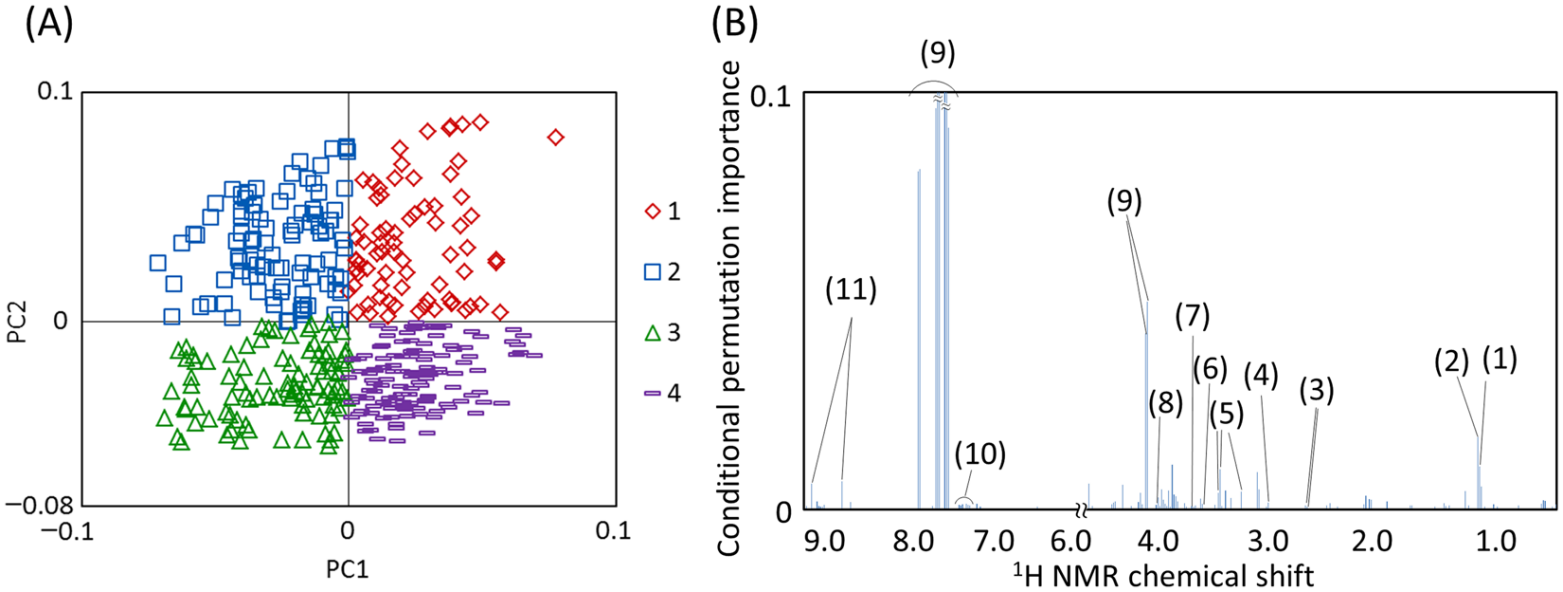
Important variables evaluated in the cforest analysis. Kernel principal component analysis results were used to generate four groups based on PC1 and PC2 plus and minus signs for the cforest analysis (**A**). The top 100 variables were depicted as important variables determined by cforest (**B**). The above-highlighted numbers correspond to metabolites as follows: 1: Methylmalonate, 2: Fucose, 3: Citrate, 4: Creatinine, 5: Taurine, 6: Glycine, 7: Ethanol, 8: Glucose, 9: Hippurate, 10: Phenylalanine, 11: Trigonelline.

**Figure 4:**
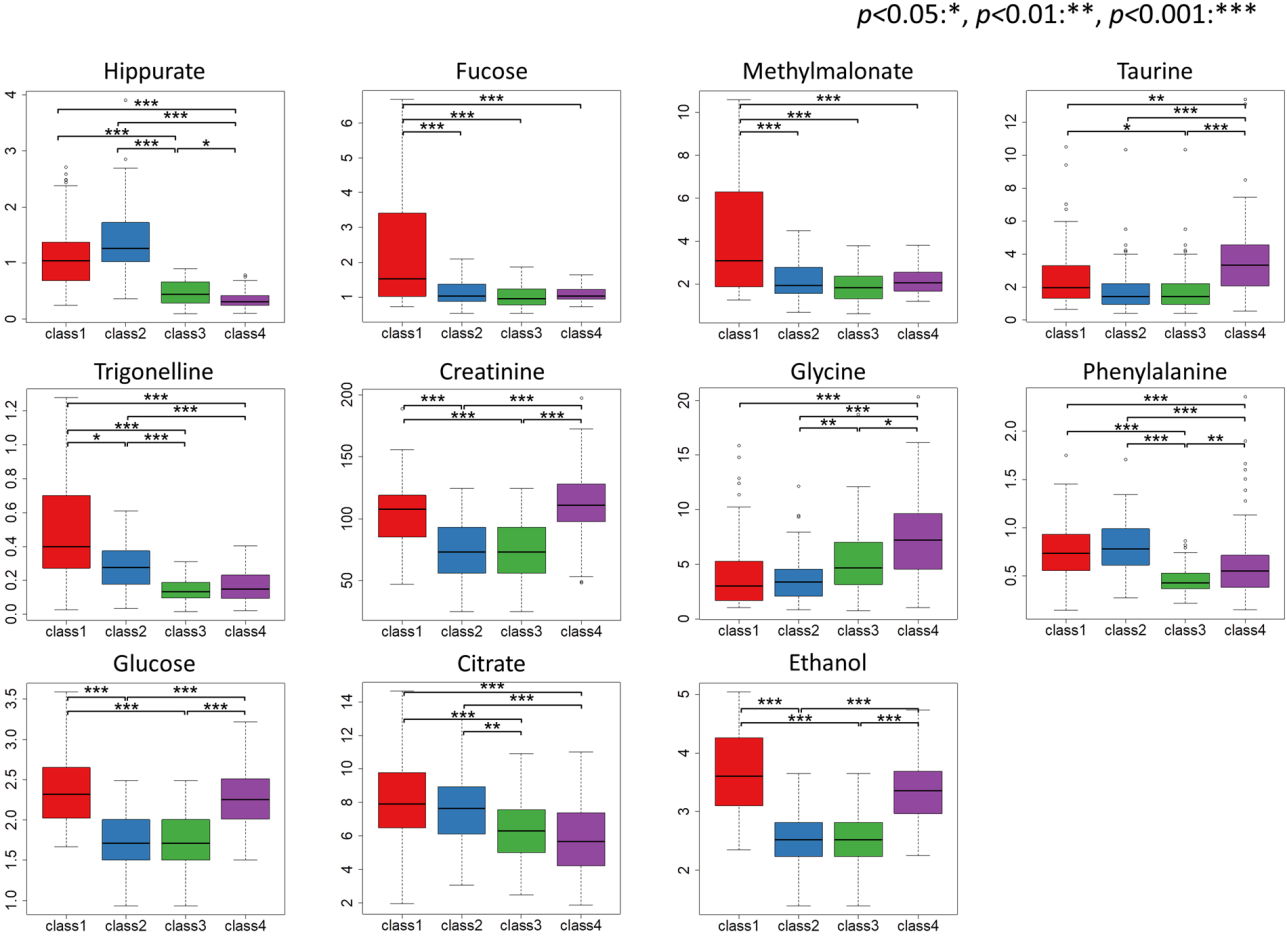
Box plots of the peak intensities of important variables. Significance: *p* < 0.05^*^, *p* < 0.01^**^, *p* < 0.001^***^.

In this study, KPCA was used for unsupervised clustering (grouping) of the dataset with no class information in a data-driven manner. The grouping is key for this analytical procedure, but KPCA cannot calculate importance of variables directly due to an inner product computation process. To overcome this limitation, we used cforest to determine the key metabolites according to the importance in a model constructed by machine learning (random forest)^34^. cforest is an unbiased tree algorithm that overcomes a major limitation of the classical random forest approach involving variable selection bias^32^. To incorporate cforest into KPCA, profiles on the KPCA score plot were mathematically classified according to principal component (PC) plus and minus signs. In the present study, four groups based on the signs of PC1 and PC2 were manually generated for the cforest analysis (Fig. 3A). In this grouping, all samples were categorized in one of the 4 classes considered in the calculation. The number of samples belonging to classes 1, 2, 3, and 4 were 73, 94, 96, and 123, respectively. The samples categorized to classes 1 and 2 had a tendency to consume vegetable and fruit diets in the previous day, whereas the samples categorized to classes 3 and 4 had a tendency to consume protein- and fat-rich diets in the previous day (Table S1). Among this tendency, the class 1 was likely to be also influenced by the alcohol intakes, and the class 3 was likely to be associated with fish intakes in the previous day. From the unsupervised grouping by KPCA in a data-driven manner, the class information was added to the original data for calculation of cforest modeling. The cforest modeling was performed with leave-one-out cross validation, resulting in 85.8% classification accuracy based on 4 classes with the confusion matrix shown in Table S2. The importance was also calculated for all variables in the cforest analysis, and the variables were aligned in descending order according to importance; only the top 100 variables are depicted in Fig. 3B. Among metabolites and inorganic elements, hippurate was identified as the most important variable, followed by fucose, methylmalonate, taurine, trigonelline, creatinine, glycine, glucose, citrate, phenylalanine, and ethanol. Significant intergroup differences were calculated to validate important variables (metabolites) (Fig. 4). For example, hippurate was significantly abundant in samples located in the positive PC2 group on the KPCA score plot compared with those in the PC2 negative group. In our previous study^35^, hippurate was detected in NMR spectra but was not focused because there was no correlation with nutritional trends (high and low protein diets) studied in the previous paper. Thus, this current study enabled to provide a different perspective (a standpoint based on hippurate) that couldn’t detect the relationship by the conventional method in the previous paper.

### Evaluation of hippurate in input–output responses

Hippurate was identified as the most important variable contributing to KPCA class information. To characterize biological contents, hippurate, other urinary metabolites and inorganic elements, and nutritional data derived from daily dietary intake records were subjected to an MBA. MBA enables to screen direct or indirect relationships though MBA manages occasionally to detect any biological irrelevant and meaningless correlations. Notably, hippurate was associated with high concentrations of some vitamins and minerals present in foods eaten during the previous day (Figure S3A). Hippurate is abundant in diets that gain a lot of nourishment from foods containing aromatic compounds (e.g., polyphenols, aromatic side-chain amino acids) such as plants^36,37^. Studies in germ-free mice, which excrete low levels of hippurate in urine, suggest that aromatic compounds from dietary components are metabolized by symbiotic gut microbiota^38^. Two other reports have described a relationship between high levels of urinary hippurate excretion and intake of certain foods including fruits, vegetables, and whole-grain wheat flour^39,40^. In our study, the intake of fruits, vegetables, and whole-grain wheat flour during the previous day was considered to indicate a high intake of nutritive components (e.g., vitamins, minerals, food fiber, and carbohydrates), and thus our observations were consistent with those of previous reports^39,40^. Additionally, fruits such as banana and citrus fruit contain aromatic compounds^41^, and hippurate excretion was found to increase over time after the consumption of orange juice^42^, a finding that was also consistent with our observation of high levels of urinary hippurate excretion following intake of edible fruits during the previous day. Overall, our data suggest that high hippurate levels observed in this study were consequent to the intake of a wide variety of vegetables and fruits.

An increasing hippurate level was also associated with increasing or decreasing levels of some output metabolites and minerals (Figure S3B). Among these metabolites and minerals, increasing levels of phenylalanine, tryptophan, and citrate have been reported to reflect the intake of whole-grain wheat flour^39^. Increasing levels of tryptophan and phenylalanine are attributed to the gut microbiotic shikimate pathway^43,44^ which resynthesize aromatic amino acids^45^.

**Figure 5:**
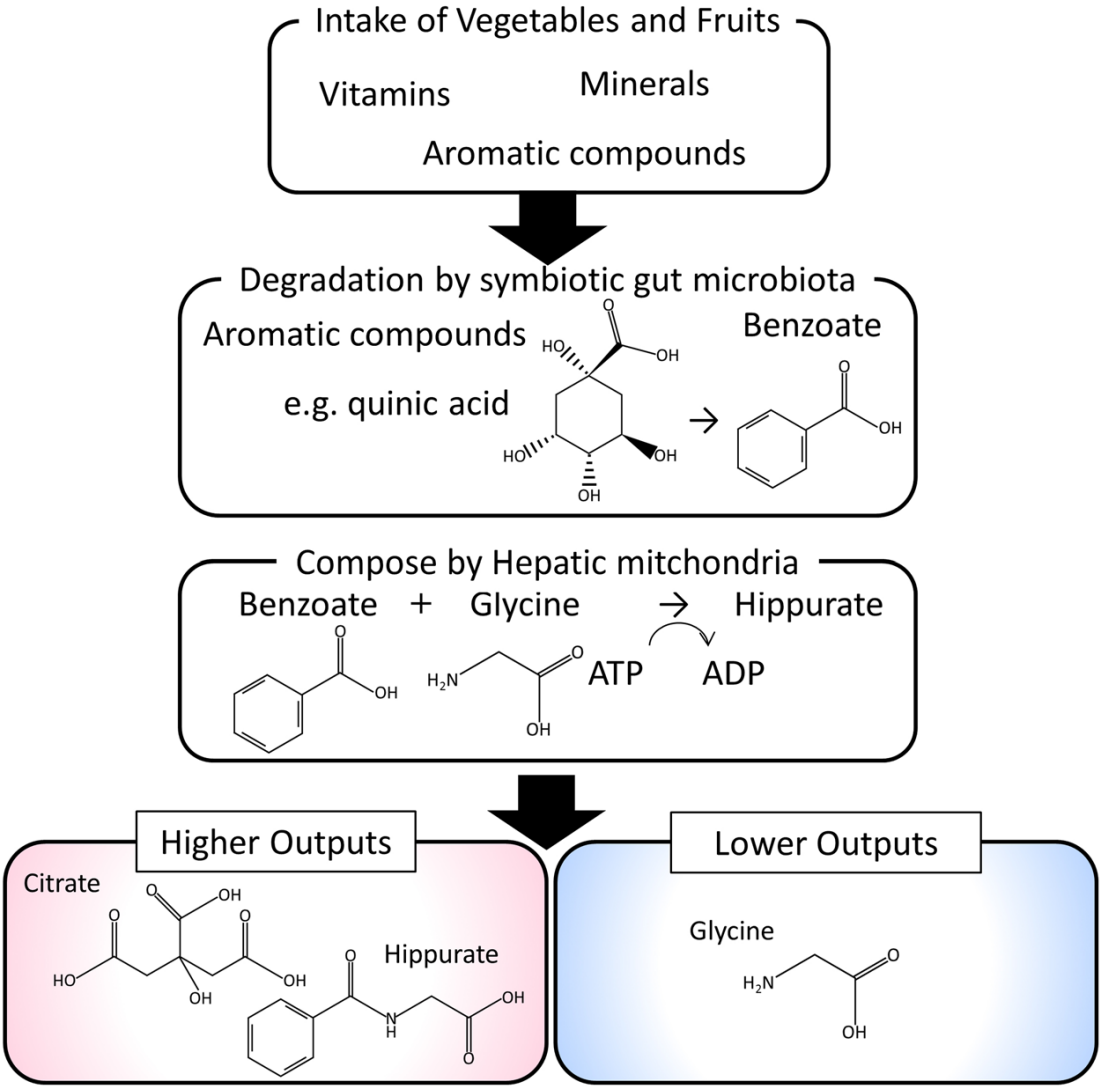
Putative pathway from dietary intake to metabolite generation. Significant metabolites were extracted using kernel principal component analysis followed by cforest, and associated intake nutrients were computed using market basket analysis.

One report suggests a relationship between reduced creatinine levels and vegetable intake^40^ as well as between hippurate production and increasing and decreasing levels of citrate and glycine, respectively. The latter is particularly relevant, as hippurate is produced from benzoate and glycine in the human liver^46^. In addition, benzoate is derived from gut microbial degradation of aromatic compounds from vegetables or fruits^37^, and citrate is an intermediate component of the TCA cycle. Hippurate production requires ATP; the increased citrate production resulting from an increased demand for ATP may explain the observed concurrent increases in hippurate and citrate levels^47^. Accordingly, the observed associations of hippurate with food intake and other metabolites were summarized into a putative simple pathway (Fig. 5). Overall, the analytical approach described here enabled us to capture input–output responses that were undetectable using linear PCA in previous studies^33,35^.

In this study, only two principle components (PC1 and PC2) were used for the categorization of KPCA in the analytical procedure because it is important to evaluate with as few components as possible in terms of dimensional (data) reduction. However, this may be not always suitable in some cases to obtain a best performance of the analytical procedure. Moreover, the grouping to 4 classes may be not always better in some cases although characteristic features in the dataset were able to be captured by the 4 class grouping with appropriate dispersion on the scores plot in this study. Therefore, it will be beneficial and effective to develop a method for automatic determination of optimal number of components and classes and to incorporate the method into the analytical procedure in the future.

To evaluate the generality and robustness of our analytical approach, a dataset obtained from skin microbiota profiling^48^ as another kind of omics data was used for the performance test. The same strategy was applied to this dataset, resulting in class information obtained from the KPCA scores plot (Figure S4A). In this categorization, the samples categorized in class 1 and in class 4 were mostly derived from the arm and from the face and neck, respectively, whereas those categorized in class 2 and in class 3 were mainly derived from the armpit, buttock, and leg (Figure S4B). The important variables contributing to each class were calculated by cforest analysis (Figure S4C), resulting in several key bacteria such as Corynebacteriaceae and Neisseriaceae identified as having high importance for the KPCA categorization. The important bacteria were further assessed by significant test among each class (Figure S5), revealing that the bacteria belonging to Corynebacteriaceae and Tissierellaceae were abundant on the arm (class 1) whereas the bacteria belonging to Propionibacteriaceae, Streptococcaceae, and Pasteurellaceae were abundantly located on the face and neck (class 4). The bacteria belonging to Moraxellaceae were relatively abundant on the armpit, buttock, and leg (classes 3 and 4) compared to the arm, face, and neck. From this analysis, our analytical approach enabled us to capture the localizations of bacteria on the body that were undetectable using linear PCA in a previous study^48^. Therefore, this approach should be useful not only for metabolic and microbiota profiling but also for profiling in other areas such as proteomics and transcriptomics in biological systems and environmental ecosystems.

## Conclusions

This study established an analytical approach based on the combined use of non-linear KPCA and cforest with validation of the extracted important variables and subsequent evaluation of detected metabolites performed by MBA to identify input–output responses in humans. This approach enabled the identification of relationships between dietary intake and metabolites that could not be detected using linear PCA. By changing the kernel functions and parameters, this novel analytical approach could potentially be applied to a wide range of analyses in which useful and valuable information is extracted from biological and environmental systems. This approach, which can be applied to non-linear trend data, should therefore be incorporated as a new analytical option in diverse fields of science (especially life sciences).

## Methods

### Data preparations

In this study, we used 386 NMR and 386 ICP-OES datasets of urine samples collected from 8 human volunteers and 309 nutritional datasets of daily dietary intake records obtained from previous studies^33,35^, and also spectral data acquired in the present study. The human ethical committees of RIKEN Yokohama Research Institute and Yokohama City University approved this study which enrolled human subjects who provided informed consent. All methods and procedures were performed in accordance with the relevant guidelines and regulations.

### Data processing

Collected ^1^H NMR data (32,000 data points) were normalized via probabilistic quotient normalization^49^ using the mQTL package (Revolution R open software, 8.0.1 beta 64-bit) and aligned using the *i*coshift^50^ program on Matlab R2015b (MathWorks Japan, Tokyo, Japan) in an in-house computing environment. Peak-picked NMR data and ICP-OES data were merged into a data matrix with auto scaling for further analyses.

### KPCA

As mentioned above, KPCA was developed for non-linear PCA^28^. Accordingly, KPCA comprises PCA, in which a non-linear kernel function has been incorporated, allowing the performance of non-linear PCA using matrices converted from input matrices by the kernel function. During processing, a kernel-enabled non-linear data approximation enables the extraction of information that differs from that obtained using conventional linear approximations. Although several methods have been developed for the kernel function, ANOVA kernel method was the best performance of dispersion on the scores plot compared to the other kernel methods, i.e., Gaussian (Figure S6), Laplace (Figure S7), and Bessel (Figure S8). Thus, the following ANOVA kernel method was used in the present study:

1 $$\text{ANOVA kernel}:\,K(x,y)={({\sum }_{(k=1)}^{n}exp(-\sigma {({x}^{k}-{x^{\prime} }^{k})}^{2}))}^{d}$$

ANOVA kernel calculations were performed using the kpca function installed in the R kernlab package^51^.

### cforest

The random forest method^34^ is a well-known machine learning algorithm for clustering and regression analyses and has become widely used in recent years in bioinformatics studies. Random forest can be applied to datasets with non-linear features and is intended for the construction of predictive and discriminant models as well as the calculation of important variables for constructing predictors. However, random forest is associated with the potential for bias caused by differences in sample numbers between groups. In other words, a group with a larger number of samples is more likely to be identified as having greater importance with respect to corresponding variables^32,52,53^. To counteract this bias, the cforest algorithm was developed based on a non-biased decision tree which overcomes the weakness associated with variable selections using conditional inference trees to calculate a permutation importance. In this study, cforest was calculated using the cforest function in the R party software package^54^ for original data with group information determined from KPCA results. In this process, we used the tuneRF function in the R randomForest package to tune ntree (number of decision trees) set to 80 and mtry (number of features used to make a decision tree) set to 900. Leave-one-out cross validation was performed for verification of the constructed model.

### Statistical analysis

MBA with NMR, ICP-OES, and nutrient variables was performed using the R software package arules^55^ as previously described^33^. The association rules were determined to exceed the cut-off values of 0.0625 for support, 0.25 for confidence, and 1.2 for lift. The association network was drawn using the Cytoscape program^56^. The Kruskal–Wallis test was used to determine significant differences between groups.

### Analytical protocol

For analysis of KPCA, the R “kernlab” package is installed from the CRAN website. Then the command “library(kernlab)” was executed for loading in the R platform. The function “kpca” was executed with kernel=“anovadot” for ANOVA function. The obtained KPCA scores were manually classified into four groups based on PC1 and PC2 plus and minus signs. Class information for four groups was added to the original data, followed by execution of the cforest program using the R “party” package installed from the CRAN website. The function “cforest” was executed, and variable importances were calculated. Finally, the variables were sorted in descending order according to their importances for further analyses such as significance tests and MBA. The R protocols (i.e., KPCA, cforest, and MBA) used in this study were deposited on our website (http://dmar.riken.jp/Rscripts/).

## Electronic supplementary material

Supporting information
