# Supplementary material for: Application of kernel principal component analysis and computational machine learning to exploration of metabolites strongly associated with diet
Source: Sci Rep. 2018 Feb 21;8:3426. doi: 10.1038/s41598-018-20121-w (PMC5821832; doi:10.1038/s41598-018-20121-w)
Supplement: Supplementary file 1 — Supporting information [file 41598_2018_20121_MOESM1_ESM.pdf]

Supporting Information

Application of kernel principal component analysis and computational machine learning to exploration of metabolites strongly associated with diet

Yuka Shiokawa[1], Yasuhiro Date[1, 2], and Jun Kikuchi[1, 2, 3, *]

[1]RIKEN Center for Sustainable Resource Science, 1-7-22 Suehiro-cho, Tsurumi-ku, Yokohama 235-0045, Japan

[2]Graduate School of Medical Life Science, Yokohama City University, 1-7-29 Suehirocho, Tsurumi-ku, Yokohama 230-0045, Japan

[3]Graduate School of Bioagricultural Sciences and School of Agricultural Sciences, Nagoya University, 1 Furo-cho, Chikusa-ku, Nagoya 464-8601, Japan

* To whom correspondence should be addressed.

Tel: +81(45)5039439. Fax: +81(45)5039489. E-mail: jun.kikuchi@riken.jp

Contents:

Figure S1. All ^{1}H NMR spectral (A) and ICP-OES (B) data (n = 386) used in this study.

Figure S2. Changes in PC1 contribution rates according to sigma. A change in sigma from 0.05 to 0.3 (above) resulted in a range expansion from 0.13 to 0.14 (below).

Figure S3. Hippurate association networks computed using market based analysis. (A) Relationships between hippurate and nutrients included in foods eaten the previous day. (B) Relationships between urinary metabolites/minerals and hippurate. Red, output metabolites and minerals; orange, input vitamins; blue, input minerals; green, input vegetables; pink, input fruits; gray, input fats; light green, input carbohydrates. "H_" indicates a high level of material; "L_" indicates a low level of material. The "high" or "low" indicated the top or the bottom 25% of all values in each urinary metabolite, urinary element, and dietary nutrient, respectively.

Figure S4. Important variables evaluated by KPCA in combination with cforest analysis for dataset of skin microbiota.[1] (A) KPCA results were used to generate four groups based on PC1 and PC2 plus and minus signs for the cforest analysis. (B) Frequency of appearance of each body part (sample category) was summarized based on each class. (C) The important variables contributing to each KPCA class were calculated by cforest. The all variables except for zero values are shown, and higher values indicate the variables identified as having high importance. UF denotes an unidentified bacterial family belonged to the higher taxon displayed in parentheses.

Figure S5. Relative abundances of each microbe detected as important variables. Significance: $p < 0.05$:*, $p < 0.01$:**, $p < 0.001$:***.

Figure S6. Evaluation of dispersion shown on PCA scores plots (PC1 vs. PC2) using the Gaussian kernel function after changing the sigma parameter from 0.00001 to 0.1 for optimization of the parameter. The scores plots were displayed in the sigma parameter of

0.00001 (A), 0.0001 (B), 0.001 (C), 0.01 (D), and 0.1 (E). The symbols and numbers indicate individual subjects.

Figure S7. Evaluation of dispersion shown on PCA scores plots (PC1 vs. PC2) using the Laplace kernel function after changing the sigma parameter from 0.001 to 1 for optimization of the parameter. The scores plots were displayed in the sigma parameter of 0.001 (A), 0.01 (B), 0.05 (C), 0.1 (D), and 1 (E). The symbols and numbers indicate individual subjects.

Figure S8. Evaluation of dispersion shown on PCA scores plots (PC1 vs. PC2) using the Bessel kernel function after changing the sigma parameter from 0.05 to 0.11 for optimization of the parameters. The scores plots were displayed in the sigma parameter of 0.05 (A, B), 0.06 (C, D, E), 0.07 (F, G, H), and 0.1 (I, J) with the parameters degree = 1 (A, C, E, F, H, I) and 2 (B, D, G, J) and order = 1 (A, B, C, D, F, G, I, J) and 2 (E, H). The symbols and numbers indicate individual subjects.

Table S1. List of association rules correlated with each class evaluated by MBA.

Table S2. Confusion matrix for evaluation of cforest performance.

Reference

1. Tsutsui, S., Date, Y. & Kikuchi, J. Visualizing Individual and Region-specific Microbial–metabolite Relations by Important Variable Selection Using Machine Learning Approaches. *Journal of Computer Aided Chemistry* **18**, 31-41 (2017).

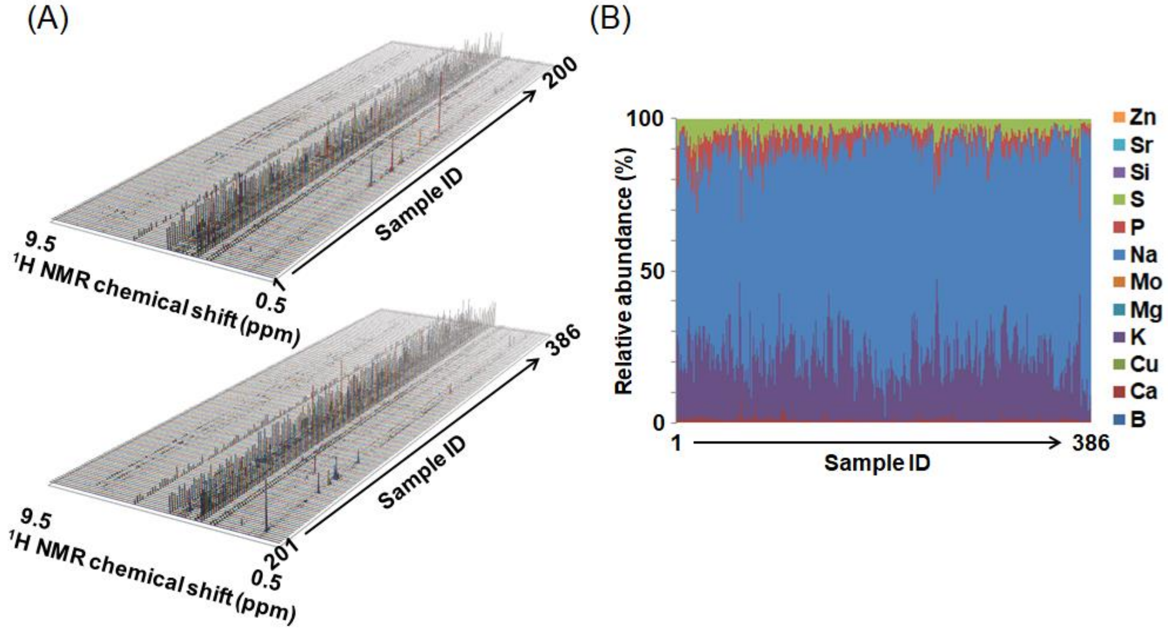

Figure S1. All ^{1}H NMR spectral (A) and ICP-OES (B) data (n = 386) used in this study.

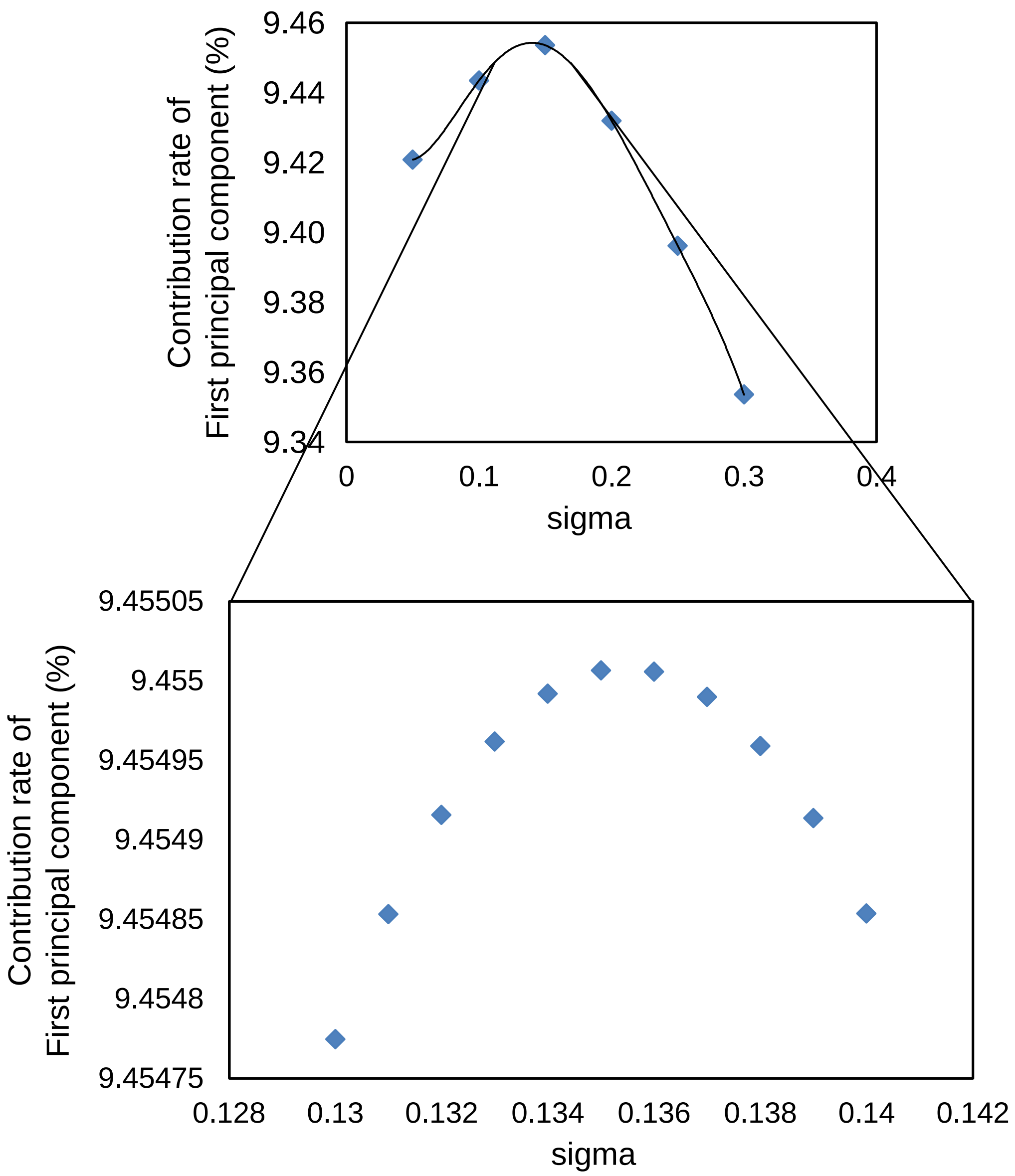

Figure S2. Changes in PC1 contribution rates according to sigma. A change in sigma from 0.05 to 0.3 (above) resulted in a range expansion from 0.13 to 0.14 (below).

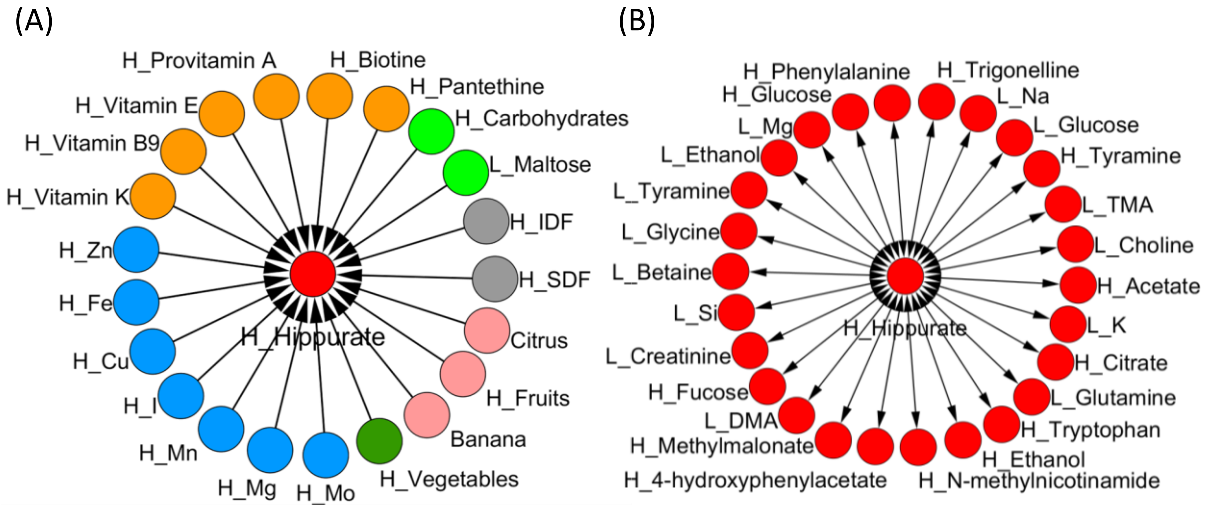

Figure S3. Hippurate association networks computed using market based analysis. (A) Relationships between hippurate and nutrients included in foods eaten the previous day. (B) Relationships between urinary metabolites/minerals and hippurate. Red, output metabolites and minerals; orange, input vitamins; blue, input minerals; green, input vegetables; pink, input fruits; gray, input fats; light green, input carbohydrates. "H_" indicates a high level of material; "L_" indicates a low level of material. The "high" or "low" indicated the top or the bottom 25% of all values in each urinary metabolite, urinary element, and dietary nutrient, respectively.

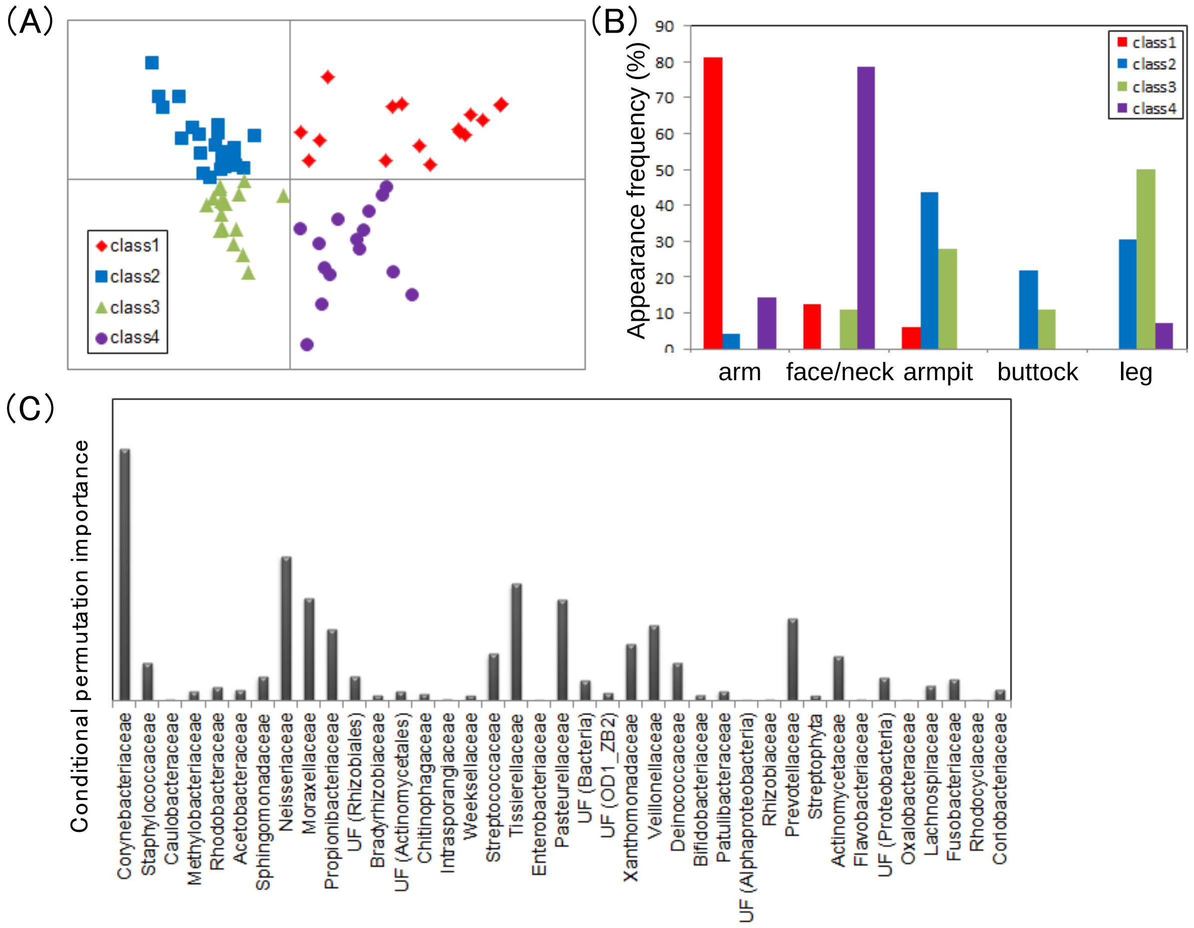

Figure S4. Important variables evaluated by KPCA in combination with cforest analysis for dataset of skin microbiota. (A) KPCA results were used to generate four groups based on PC1 and PC2 plus and minus signs for the cforest analysis. (B) Frequency of appearance of each body part (sample category) was summarized based on each class. (C) The important variables contributing to each KPCA class were calculated by cforest. The all variables except for zero values are shown, and higher values indicate the variables identified as having high importance. UF denotes an unidentified bacterial family belonged to the higher taxon displayed in parentheses.

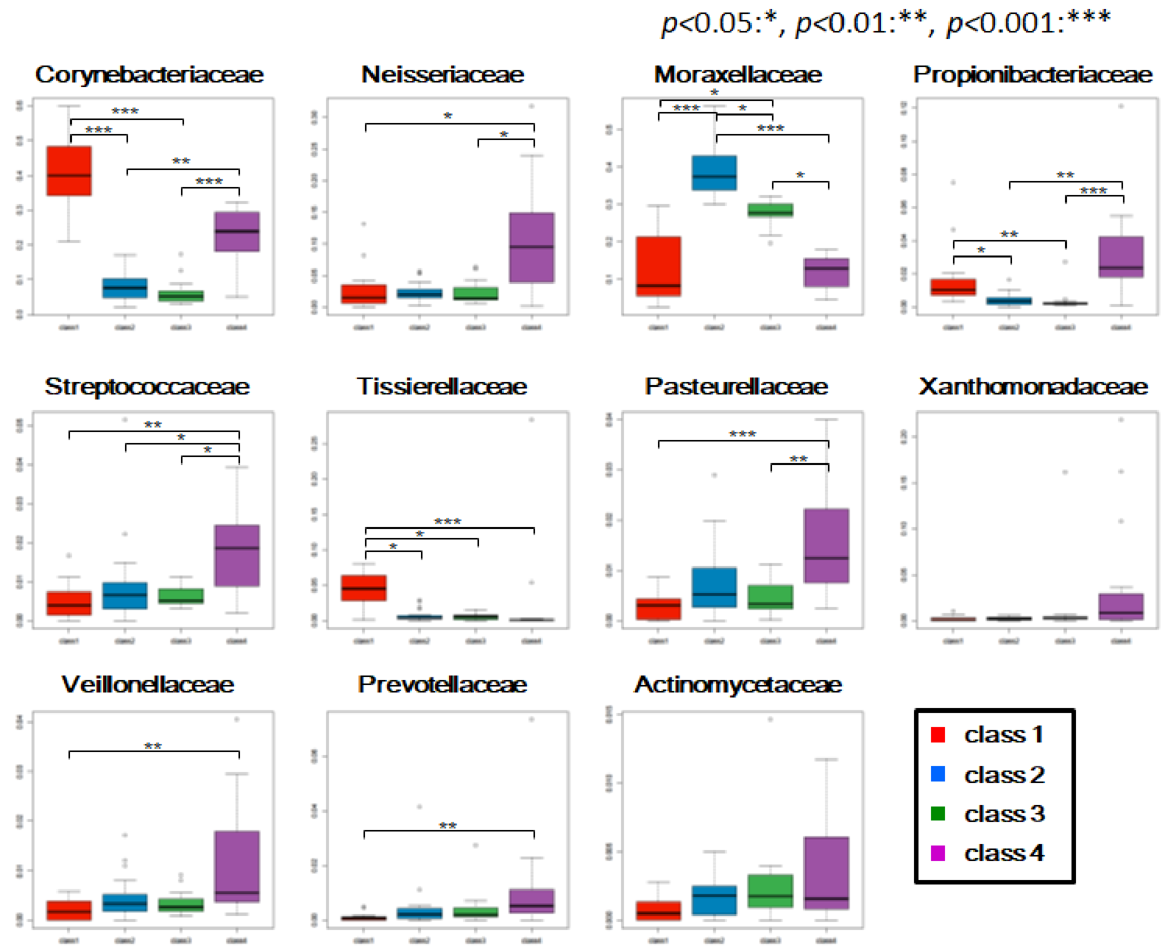

Figure S5. Relative abundances of each microbe detected as important variables. Significance: $p < 0.05$:*, $p < 0.01$:**, $p < 0.001$:***.

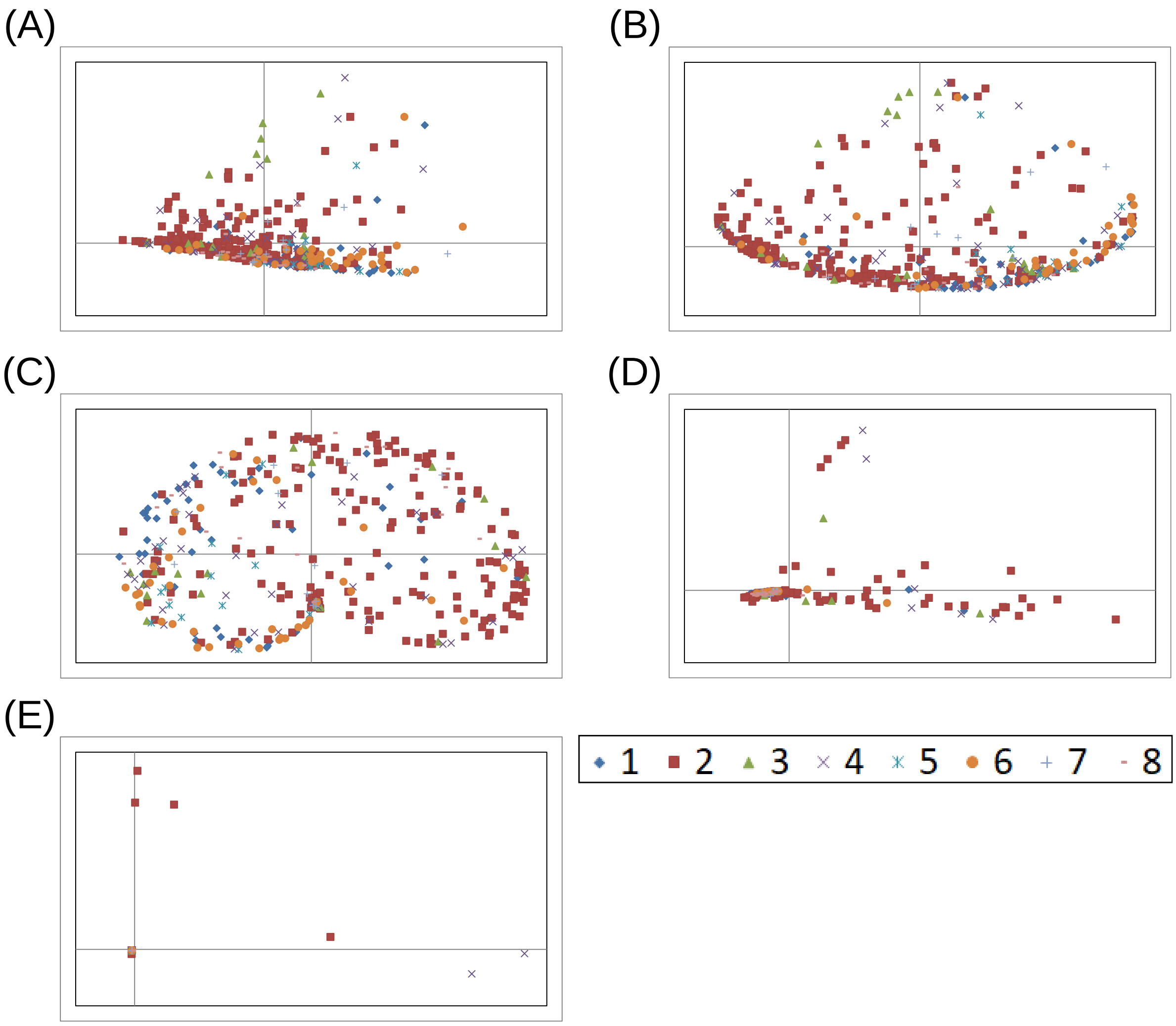

Figure S6. Evaluation of dispersion shown on PCA scores plots (PC1 vs. PC2) using the Gaussian kernel function after changing the sigma parameter from 0.00001 to 0.1 for optimization of the parameter. The scores plots were displayed in the sigma parameter of 0.00001 (A), 0.0001 (B), 0.001 (C), 0.01 (D), and 0.1 (E). The symbols and numbers indicate individual subjects.

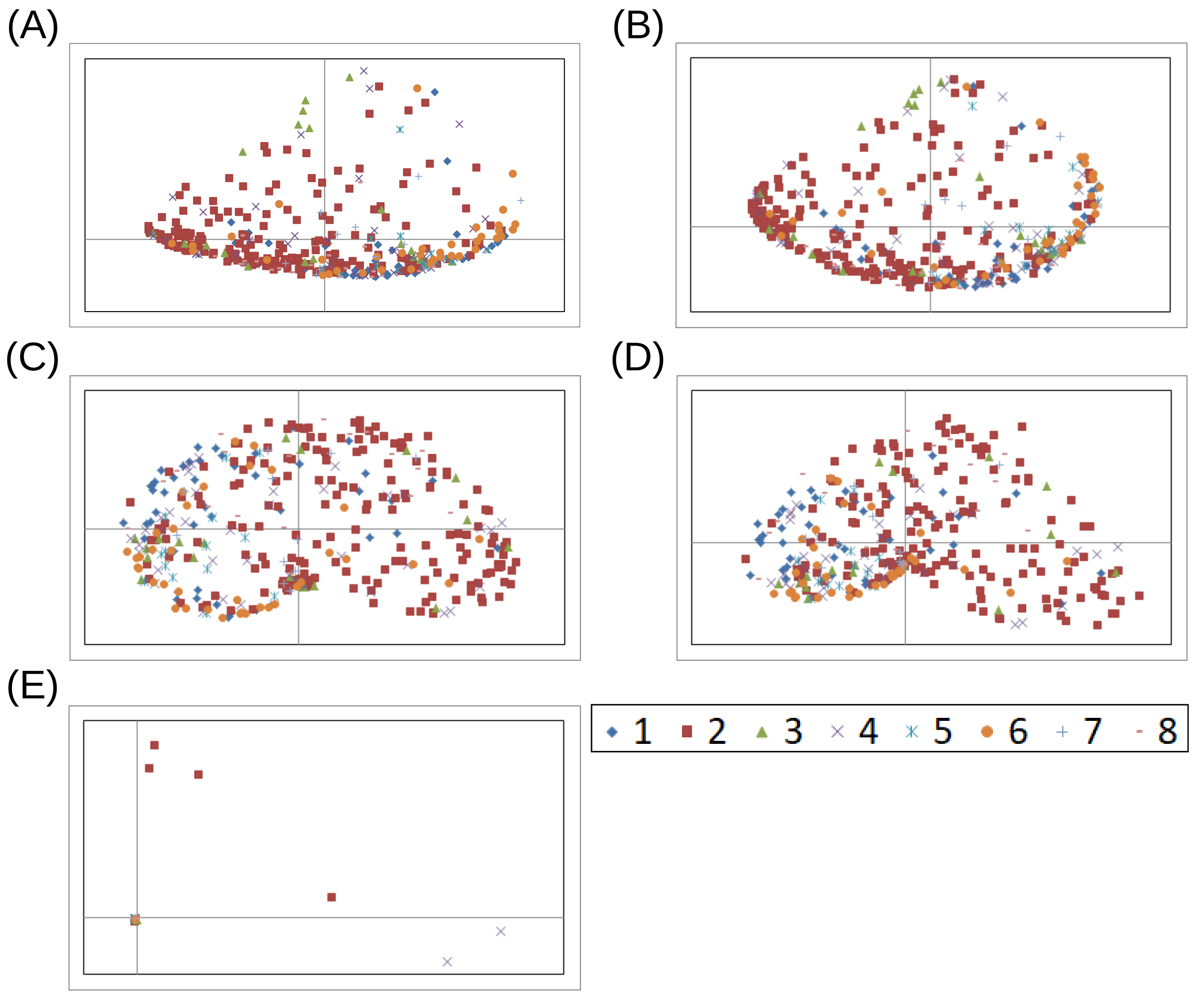

Figure S7. Evaluation of dispersion shown on PCA scores plots (PC1 vs. PC2) using the Laplace kernel function after changing the sigma parameter from 0.001 to 1 for optimization of the parameter. The scores plots were displayed in the sigma parameter of 0.001 (A), 0.01 (B), 0.05 (C), 0.1 (D), and 1 (E). The symbols and numbers indicate individual subjects.

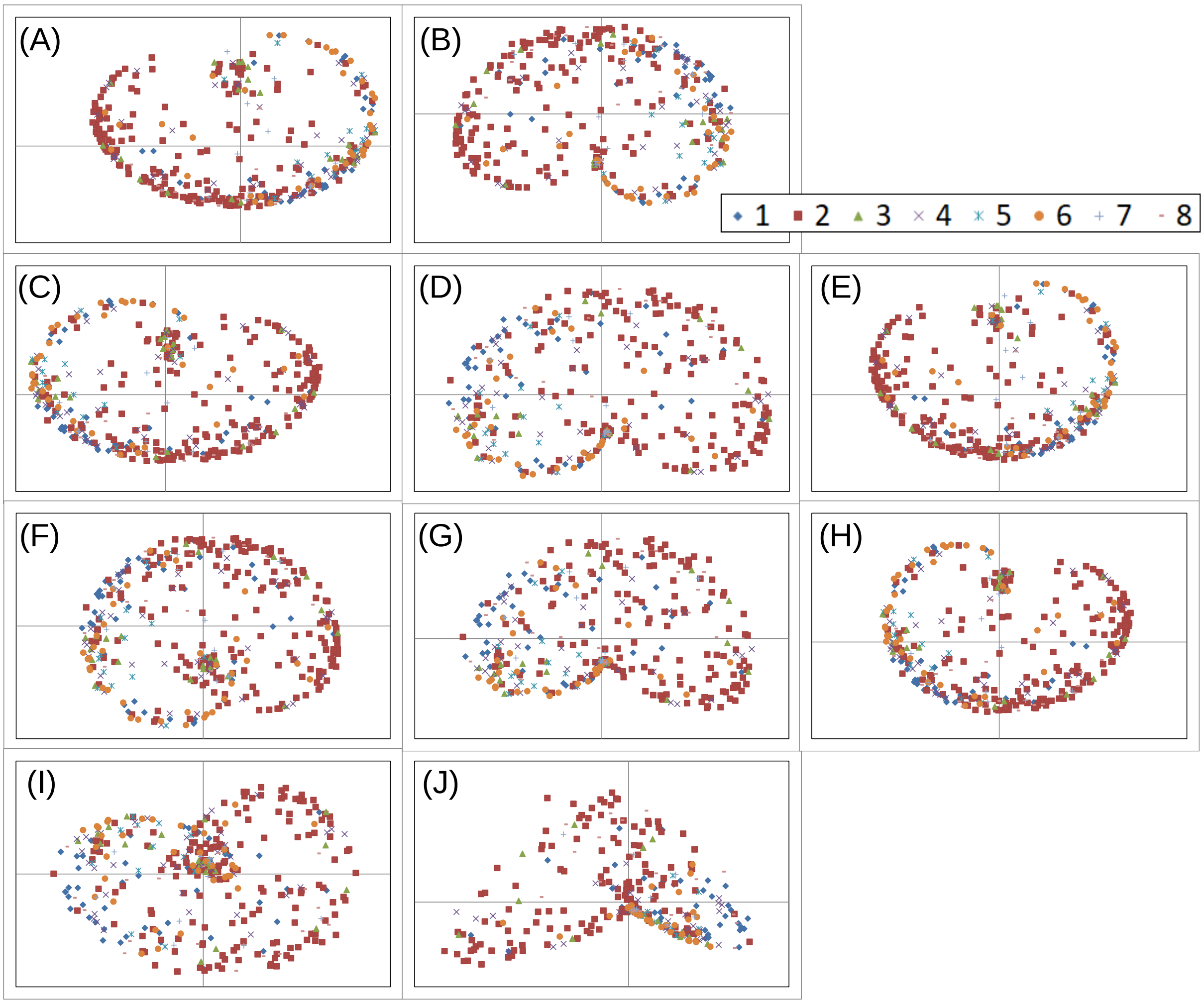

Figure S8. Evaluation of dispersion shown on PCA scores plots (PC1 vs. PC2) using the Bessel kernel function after changing the sigma parameter from 0.05 to 0.11 for optimization of the parameters. The scores plots were displayed in the sigma parameter of 0.05 (A, B), 0.06 (C, D, E), 0.07 (F, G, H), and 0.1 (I, J) with the parameters degree = 1 (A, C, E, F, H, I) and 2 (B, D, G, J) and order = 1 (A, B, C, D, F, G, I, J) and 2 (E, H). The symbols and numbers indicate individual subjects.

Table S1. List of association rules correlated with each class evaluated by MBA.

Association rules			lift value
Alcohol	=>	class 1	1.66
Vitamin D	=>	class 1	1.47
Vegetables	=>	class 1	1.40
Fatty acids	=>	class 1	1.38
Fructose	=>	class 1	1.28
Citrus	=>	class 2	2.15
Fruits	=>	class 2	2.05
Fe	=>	class 2	2.01
Vitamin B9	=>	class 2	1.88
Soluble dietary fiber	=>	class 2	1.84
Pantetine	=>	class 2	1.81
Mo	=>	class 2	1.79
Provitamin A	=>	class 2	1.73
Insoluble dietary fiber	=>	class 2	1.68
Vitamin E	=>	class 2	1.60
Vegetables	=>	class 2	1.58
Mg	=>	class 2	1.57
Vitamin K	=>	class 2	1.55
Banana	=>	class 2	1.54
Carbohydrates	=>	class 2	1.42
I	=>	class 2	1.42
Zn	=>	class 2	1.35
Cu	=>	class 2	1.29
Starch	=>	class 2	1.29
Fish	=>	class 3	1.49
Banana	=>	class 3	1.38
Vitamin B12	=>	class 3	1.28
Polyunsaturated fatty acids	=>	class 3	1.23
n-6 Polyunsaturated fatty acids	=>	class 3	1.21

*The cutoff values of the MBA are 0.0625 for support, 0.25 for confidence, and 1.2 for lift.

**It is displayed only the rules that each class was associated with high concentrations of nutrients present in foods eaten during the previous day.

Table S1. Continued.

Association rules			lift value
Lactose	=>	class 4	1.56
Cystine	=>	class 4	1.51
Glutamate	=>	class 4	1.50
Proline	=>	class 4	1.47
Saturated fatty acids	=>	class 4	1.42
Histidine	=>	class 4	1.38
Starch	=>	class 4	1.38
βCarotene	=>	class 4	1.35
Sulfur amino acid	=>	class 4	1.35
Glycine	=>	class 4	1.33
Phenylalanine	=>	class 4	1.33
Methionine	=>	class 4	1.31
Aromatic amino acid	=>	class 4	1.30
Tryptophan	=>	class 4	1.30
Branched chain amino acids	=>	class 4	1.30
Leucine	=>	class 4	1.28
Tyrosine	=>	class 4	1.28
Arginine	=>	class 4	1.25
Serine	=>	class 4	1.25
Amino acids	=>	class 4	1.24
Monounsaturated fatty acids	=>	class 4	1.23
Isoleucine	=>	class 4	1.22
Vitamin A	=>	class 4	1.21
Vitamin B2	=>	class 4	1.21
Vitamin B3	=>	class 4	1.21
Protein	=>	class 4	1.20

*The cutoff values of the MBA are 0.0625 for support, 0.25 for confidence, and 1.2 for lift.
**It is displayed only the rules that each class was associated with high concentrations of nutrients present in foods eaten during the previous day.

Table S2. Confusion matrix for evaluation of cforest performance.

observed	predicted			
	class 1	class 2	class 3	class 4
class 1	80.8	2.7	1.4	15.1
class 2	8.5	85.1	5.3	1.1
class 3	0.0	4.2	83.3	12.5
class 4	2.4	0.0	6.5	91.1
